# Design and Optimisation of an Enterprise Digital Management System Based on IoT Monitoring

**DOI:** 10.1155/2022/3264485

**Published:** 2022-06-24

**Authors:** Shengwen Wang

**Affiliations:** School of Business Administration, Shandong University of Finance and Economics, Jinan 250014, China

## Abstract

Modern enterprise management is developing rapidly, and one of the most important components of enterprise management is enterprise digital management. In this paper, the research background of enterprise digital management system is firstly elaborated, current situation of enterprise digital management work in enterprises is elaborated, then the current situation of the system research and development at home and abroad is also analysed and outlined in detail, and finally, according to the actual enterprise digital management situation of enterprises, combined with the system method of software development and corresponding technology, the new system design requirements of enterprises based on Internet of Things are proposed. Simulations show that the overall design and module design of the system in this paper are well managed, a system interface diagram can be given, and the database table structure and entity class diagram are applied to elaborate the database design.

## 1. Introduction

Digital management is an important aspect of the management of modern enterprises, and the survival and development of enterprises is closely related to strengthening the protection of their digital resources [1]. Enterprises have been using traditional management methods for a long time, manually locating and retrieving digital files, and designing and printing hard copies of digital catalogues [2]. However, this traditional management approach requires human participation, leading to a variety of problems such as inefficiency and difficulty storing and securing documents [3]. In the course of an enterprise's daily operations, large numbers of digital documents are generated at all levels, and these documents all need to be archived [4]. The archiving of corporate documents is generally not undertaken in a timely manner. To meet the management requirements of modern enterprises, a digital enterprise management system should consist of six parts: scanning and entry, digital management, enquiry and statistics, loan management, approval management, and system management [5]. The rapid development of Internet-based information technology systems has resulted in enterprises attaching increasing importance to digital information management [6]. The use of Internet technology is an important aspect of a digital information system, while the application of digital information resources provides support for the management and transformation of enterprises [7]. Therefore, it is necessary for each enterprise to build a digital management system that meets its specific requirements and enables it to keep up with the trend of increasing informatisation [8]. This study is based on the integration of the main activities of an enterprise with advanced management information system technology, and aims to improve the efficiency of enterprise management by designing a digital enterprise management system [9]. Based on the needs of enterprises, the digital enterprise management system is designed to facilitate information exchange and communication that overcomes problems such as poor information communication caused by inconvenient locations, incompetence, and operational distance, which can hinder the development of the enterprise [10, 11].

## 2. RFID-Based Enterprise Digital Management Platform Architecture

**Figure 1:**
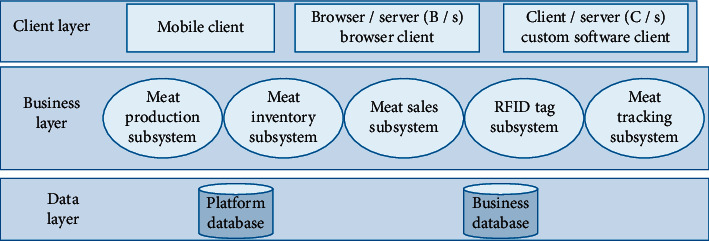
RFID-based enterprise digital management platform architecture.

The architecture of the RFID-based enterprise digital management platform is shown in Figure 1 and is divided into a data layer, a business layer, and a customer layer. The data layer includes the platform database and the business database. The platform database provides the operational data of the basic platform, and the business database provides the business data [11]. The business layer provides the main functions of the system, which mainly consists of five subsystems: RFID tag subsystem, enterprise production subsystem, enterprise inventory subsystem, enterprise sales subsystem, and enterprise tracking and tracing subsystem. The client layer provides the interface for users to use the system in different ways, mainly divided into mobile phone clients, browser clients under the browser/server (B/S) structure, and custom software clients under the client/server (C/S) structure. Users are divided into 3 main categories: consumers, administrators, and supervisors.

The EPC system is mainly responsible for the collection and filtering of RFID data from each subsystem node, which is transmitted via the network to the respective remote PML database for processing such as storage and query.

The lifecycle of an enterprise, from slaughter to the consumer, can be divided into several stages: slaughter, processing, inventory, transport, sales, and consumption. To achieve enterprise tracking and tracing, it is necessary to identify, record, manage, and track the information in these stages to form a complete enterprise lifecycle information chain [12]. Among them, the enterprise information tracking and tracing in the slaughtering and processing stage has already been discussed in this paper. This paper focuses on the tracking and tracing of information in the sales phase of the enterprise.

### 2.1. Analysis of Corporate Sales Tracking Systems

In a radio frequency identification (RFID)-based information system environment, the volume of information is so large that efficient RFID event detection and processing is difficult to achieve using existing database and data management technologies [14]. This system uses the stand-alone synchronisation equipment (SASE) event language of complex event processing (CEP) technology to process the basic events formed by the filtered data [15]. CEP is an emerging technology for building and managing information systems [16]. CEP defines the basic information to be processed as events, which can be customised by the user, for example, as an event related to an operation in an enterprise application or as an event related to a data transfer on the network, depending on the user's role and observation point [17]. There are various relationships between simple events, and multiple simple events can be combined to form a complex event. Further, multiple complex events can be combined to form a “more complex” event. The process of using CEP to analyse data is as follows. First, the layout of the RFID reader is designed based on the requirements of each node in the enterprise's operations. Second, basic events are defined based on the layout of the RFID reader. Finally, complex events are processed using the SASE event language [18, 19].

### 2.2. Operating mechanism of corporate sales tracking systems

**Figure 2:**
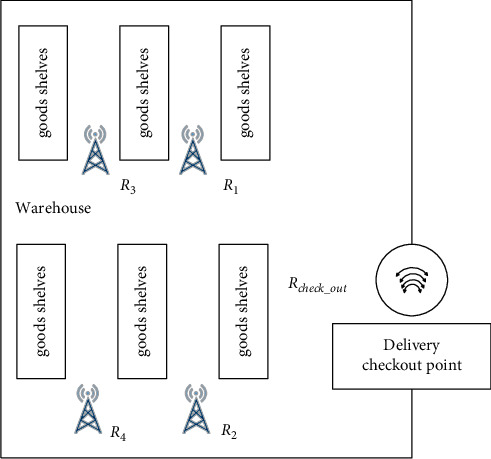
Layout of RFID readers in the sales centre warehouse.

The following is an example of RFID data analysis of an enterprise's inventory node to illustrate the process of RFID data analysis using CEP. 1) The enterprise's regional sales centre is the main link in the circulation of the enterprise's products and requires information about the enterprise's sales, stock, and expired products. Based on an analysis of the functions required by the enterprise's regional sales centre, it is clear that the information to be collected includes stock stored in the enterprise's warehouse, sales through the regional sales centre, and expired stock in the enterprise's warehouse. Therefore, it is necessary to position RFID readers inside the warehouse, at the warehouse exit, and at the warehouse settlement point, as shown in Figure 2. 2). The three basic events in the warehouse inventory node are WAREHOUSE-READING, CHECK_OUT-READING, and EXIT-READING, where WAREHOUSE-READING represents the data from the RFID readers (R1, R2, R3, R4 in Figure 2) in the warehouse, CHECK_OUT-READING represents the data from the tags read by the checkout reader (Rcheck_out in Figure 2 when the enterprise ships stock, and EXIT-READING represents the data read by the warehouse exit reader (Rcheck_out in Figure 2 when the stock leaves the warehouse [20, 21] 3) Regarding the processing of complex events in the warehouse inventory node, the following is an example of how the SASE event language is processed. The complex event of enterprise sales is represented in the sales centre node model as follows. The stock arrives at the shipping checkout point and then arrives at the warehouse door, which is represented in the basic event set as a sequence of two basic events, CHECK_OUT- READING and EXIT-READING, and is completed within a defined time frame. When this complex event occurs, its EPC code and exit time are provided and this information is stored in the product exit table of the event database. In conjunction with the example shown in Figure 4, the complex event processing in relation to the sale of this stock involves checking that a label with the EPC code 86.0257A08.100167.20100608432 appears in sequence in the two basic events CHECK_OUT-READING and EXIT-READING with a time interval of less than 1 h. If this is the case, the enterprise is considered to have completed the sale of the stock.

## 3. Establishment of Decision Support Systems

**Figure 3:**
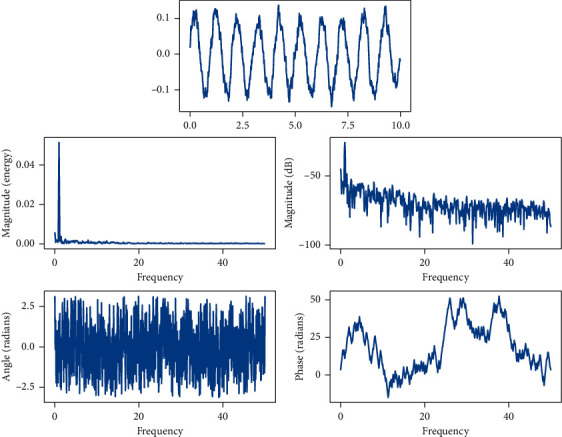
Frequency of use of different data information.

When the information accumulated by the system reaches a certain scale, advanced data mining technology can be used to intelligently identify information with potential utility from the data warehouse and provide decision support information to the leaders of relevant departments for the purpose of effective accident prevention. The system is designed with decision support interfaces included in both the data layer and the key service layer of the software. The decision support system is an independent subsystem, the overall structure of which is shown in Figure 3. The main functions of the decision support system are as follows. (1)Safety problem causation analysis for decision-making. The safety problem causation analysis decision support system assists managers in the analysis of the causes of safety problems and the assignment of responsibility for those problems. It analyses the internal and external environment in which safety problems arise, uses multidimensional analysis techniques to study the intrinsic connection of safety data, compares problems with similar problems that have occurred in the past, and completes a report on the causes of problems through human-computer interaction. (2) Accident prediction and decision-making. Predictive analysis is carried out using theories and methods such as fuzzy diagrams, grey systems, nonlinear regression, stochastic processes, and safety system engineering. Correlations between factors affecting safe production and safety problems during production are obtained [19]. The applicability of various prediction methods is examined so that the results produced by the model provide a scientific basis for the prevention and management of safety problems. (3) Research on safety countermeasures. Safety information contains a wide variety of problem types. When the number and severity of problems are within specified limits, general management measures can be applied. However, when they reach or exceed those limits, safety improvements are required. This requires the identification of problem-prone units, lines, or sections, as well as potentially problematic units, lines, or sections, and then the application of appropriate countermeasures. This mainly includes the identification of accidents, daily safety management countermeasures and safety engineering improvement countermeasures. (4) Safety warning mechanism. This involves the establishment of an assessment mechanism based on conditions such as problem level and unit type, which is then combined with historical data to enable a scientific assessment of the production safety status of each unit. A safety early warning mechanism is also established and regular safety early warning notices are issued in a targeted manner with the aim of preventing seasonal accidents.

## 4. Simulation Implementation

**Figure 4:**
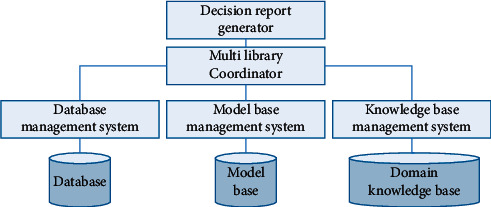
Overall structure of the decision support system.

**Figure 5:**
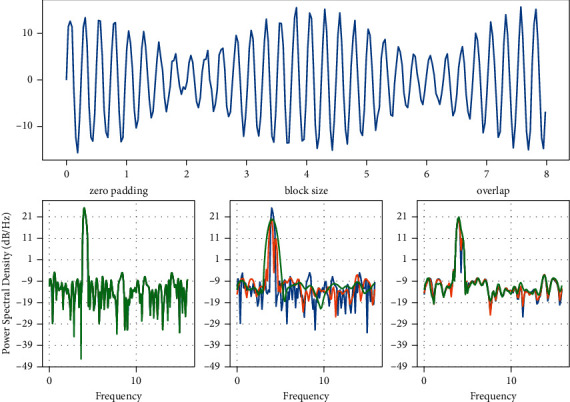
Effect of using company documents.

**Figure 6:**
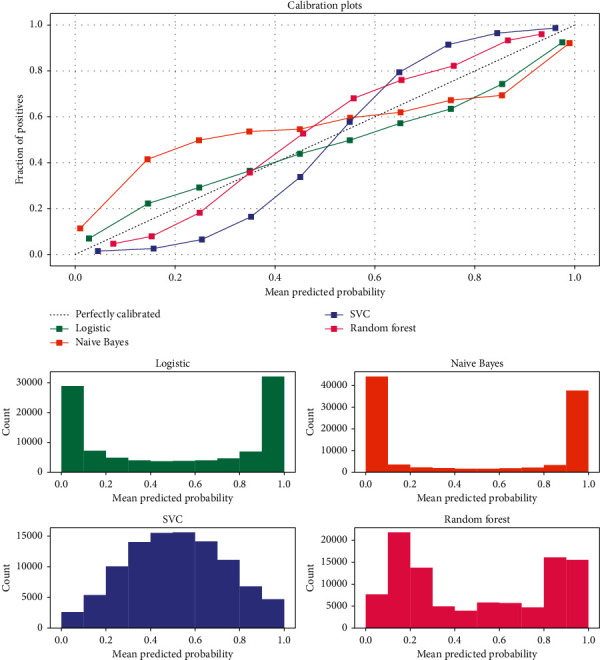
Effectiveness of digital management of companies with different solutions.

In summary, the RFID/GIS-based enterprise sales tracking and tracing system, which is a sub-system of the enterprise digital management platform architecture, needs to be integrated with other subsystems to enable the sharing of information. The frequency of data usage by the enterprise's employees is shown in Figure 4. Using the EPC codes of the products the enterprise buys, inputting these into the system through the reader on the traceability terminal and clicking on the product traceability function, it is possible to obtain the product circulation path from the manufacturer to the retailer. The relevant information regarding each product circulation node can be obtained from the GIS system. The digital enterprise management platform features hierarchical architecture containing a customer layer, a business layer, and a data layer. It is feasible to develop an enterprise sales tracking and tracing system using a unified enterprise digital management platform with hierarchical architecture. The enterprise sales link, which includes storage, transportation, sales, and other processes, does not require sales to be divided or combined with other activities, and is particularly suitable for the use of RFID tags. The system design proposed in this study was verified using enterprise documents, as shown in Figure 5. In the life-cycle management of the enterprise, based on the sales process key node RFID data collection and processing, the electronic product code system is used to develop an Internet of Things environment for enterprise information tracking and tracing. However, the system needs to ensure the validity of the data flow, and each key node in the business process must be connected to the entity tag language server of the producer to obtain the necessary information. If the data from one link cannot be updated, the integrity of the tracking and tracing information cannot be guaranteed. The effectiveness of various digital enterprise management solutions is shown in Figure 6. The good results obtained using the model proposed in this study can be attributed to the use of HF/UHF RFID technology, process optimisation and database backend control to ensure that the information is correctly collected and filtered. How to ensure stable operation of the equipment and fast, accurate data collection and processing is a challenging problem that requires further research.

## 5. Conclusions

In this study, we developed an enterprise information tracking and tracing platform based on GIS using three-tiered architecture. This platform is required to support multiuser and multiterminal devices, and thus requires the installation of the necessary GIS software in different terminals. The model proposed in this study uses process optimisation, database backend data control, and other measures to ensure that the data is safe and secure. However, the best way to ensure the security of information is to focus on the security of the RFID tags used in the front-end solution, which requires further research.

## Data Availability

The experimental data used to support the findings of this study are available from the corresponding author upon request.
